# Chromatic Perceptual Learning but No Category Effects without Linguistic Input

**DOI:** 10.3389/fpsyg.2016.00731

**Published:** 2016-05-25

**Authors:** Alexandra Grandison, Paul T. Sowden, Vicky G. Drivonikou, Leslie A. Notman, Iona Alexander, Ian R. L. Davies

**Affiliations:** ^1^School of Psychology, University of SurreyGuildford, UK; ^2^Nuffield Laboratory of Ophthalmology, University of OxfordOxford, UK

**Keywords:** color, perceptual learning, plasticity, categorical perception, lateralization

## Abstract

Perceptual learning involves an improvement in perceptual judgment with practice, which is often specific to stimulus or task factors. Perceptual learning has been shown on a range of visual tasks but very little research has explored chromatic perceptual learning. Here, we use two low level perceptual threshold tasks and a supra-threshold target detection task to assess chromatic perceptual learning and category effects. Experiment 1 investigates whether chromatic thresholds reduce as a result of training and at what level of analysis learning effects occur. Experiment 2 explores the effect of category training on chromatic thresholds, whether training of this nature is category specific and whether it can induce categorical responding. Experiment 3 investigates the effect of category training on a higher level, lateralized target detection task, previously found to be sensitive to category effects. The findings indicate that performance on a perceptual threshold task improves following training but improvements do not transfer across retinal location or hue. Therefore, chromatic perceptual learning is category specific and can occur at relatively early stages of visual analysis. Additionally, category training does not induce category effects on a low level perceptual threshold task, as indicated by comparable discrimination thresholds at the newly learned hue boundary and adjacent test points. However, category training does induce emerging category effects on a supra-threshold target detection task. Whilst chromatic perceptual learning is possible, learnt category effects appear to be a product of left hemisphere processing, and may require the input of higher level linguistic coding processes in order to manifest.

## Introduction

Color is an important aspect of our visual environment and it is argued that the human visual system has a higher sensitivity to color than any other visual stimulus (Chaparro et al., 1993). Since the work of Hering (1878, 1964) and Helmholtz (1896, 1925), human color vision has been one of the most widely investigated topics. Despite this, there is still much that remains unknown about human color vision. It is widely accepted, however, that color vision is categorical in nature. Although the color spectrum is a physical continuum of light, we perceive it as distinct bands or categories (Harnad, 1987; Goldstone and Hendrickson, 2010). The process of categorization is fundamental to human cognition as it enables us to process information efficiently. Categorization is used as a functional strategy in a large number of different domains including color. Due to the significance of color vision and the salience of categorization, there is a plethora of work exploring color categorization from a range of different perspectives (e.g., Berlin and Kay, 1969/1991; Bornstein et al., 1976; Hardin and Maffi, 1997; Yendrikhovskij, 2001). Evidence from research into color categorical perception (whereby stimuli that fall either side of a category boundary are discriminated more quickly and accurately than equally different stimuli from the same category) suggests that color perception changes as a result of experience (e.g., Roberson et al., 2000; Franklin et al., 2008b). It is the perceptual learning of color categories that is of central interest in the current investigation.

Perceptual learning is characterized by improvement in perceptual judgment following a period of practice. Research on perceptual learning has shown that practice can lead to improved performance on a range of visual tasks such as identifying orientation (Schoups et al., 2001), discriminating gratings (Fine and Jacobs, 2000), textures (Karni and Sagi, 1991) and motion (Zanker, 1999), and detecting luminance contrasts (Sowden et al., 2002) and tone contrasts (Ortiz and Wright, 2009). The effects of perceptual learning within the context of visual categorization have also been explored (e.g., Goldstone, 1994; Livingston et al., 1998; Stevenage, 1998; Notman et al., 2005; Hendrickson et al., 2012; Little et al., 2012). Perceptual learning is frequently found to be particular to stimulus or task specificities (Lu et al., 2011). For example, the effectiveness of perceptual learning has been found to vary with manipulations of feedback (see Dosher and Lu, 2009), variations in the number of trials used (Censor and Sagi, 2009) and the type of training schedule (Xiao et al., 2008). Indeed, Lu et al. (2011) suggest that understanding the factors that determine the transfer of learning is a key challenge within the domain of perceptual learning. Whilst there are a number of studies that document perceptual learning and the mechanisms that contribute to this process, there have been few studies exploring perceptual learning in the domain of color (for exceptions see Özgen and Davies, 2002; Zhou et al., 2010; Kwok et al., 2011; Clifford et al., 2012).

Özgen and Davies (2002) showed that it is possible to induce color categorical responding on a same-different task following training on a new category boundary situated in the center of an existing linguistic category. These findings provide evidence for the plasticity of color discrimination and the malleability of the structure of color categories. However, from these findings alone, the mechanisms that are responsible for acquired color category effects are unclear. It is possible that learning occurred as a result of changes in perceptual sensitivity induced by category training. However, it is also possible that cognitive processes such as memory or language contributed to these effects. For example, Clifford et al. (2012) using an event-related potential (ERP) visual oddball task, found that learned categorical effects were only related to post-perceptual ERP components, indicating that acquired color category effects are mediated by cognitive rather than perceptual mechanisms on a task of this nature. Similarly, delayed tasks such as the same-different judgment task used by Özgen and Davies (2002) are vulnerable to memory processes, and so it is likely that participants relied on a memory trace for the first stimulus when judging whether the second stimulus was the same or different (see Suegami et al., 2014). It is also possible that, in line with Linguistic Relativity, linguistic strategies enhance performance on a same-different judgment task such as this (e.g., Winawer et al., 2007). During training, stimuli may become associated with appropriate category labels, which are later accessed when the stimuli are presented. The use of category labels acts as a way of assigning meaning to perceptually unfamiliar stimuli, which has been found to improve visual processing (Lupyan and Spivey, 2008). In agreement with this possibility, chromatic thresholds do not show evidence of categorical perception at color category boundaries, which may be because threshold tasks do not access the verbal labels that result in categorical responding (Danilova and Mollon, 2009; Roberson et al., 2009). However, Kwok et al. (2011) used a perceptual learning paradigm to reveal that the acquisition of new color categories increases the volume of gray matter in the cortex. Participants undertook 2 h training across 3 days. Magnetic resonance imaging (MRI), used before and after training, showed that gray matter increased in visual area V2/3. This suggests that learning new color categories can result in perceptual learning that is accompanied by change in early stages of visual processing. One complicating factor, is that all of the training tasks used by Kwok et al. (2011) made explicit use of category labels during training, which has been found to guide the visual learning process (see Lupyan and Spivey, 2010).

Thus, the current study seeks to investigate the circumstances under which chromatic perception can change and whether such changes could underpin categorical color perception. We use a novel approach combining three different experiments to build on the growing body of work in this domain. First, in Experiment 1, we explore whether threshold training induces changes in chromatic thresholds. We use these behavioral data to make inferences about the likely neural locus of these changes. Second, in Experiment 2 we explore whether learning a new color category can drive changes in chromatic thresholds and whether changes in thresholds result in categorical perception. Finally, in Experiment 3, to test the role of possible linguistic influences we explore whether learning a new color category results in categorical color perception as measured using a lateralized supra-threshold chromatic target detection task.

## Experiment 1: Transfer of Chromatic Perceptual Learning Across Hue and Location

Experiment 1 directly tests whether chromatic perception can change as a result of training and explores the potential neural loci of these changes. Previous work with achromatic stimuli has found that perceptual learning of a wide range of stimuli is often specific to dimensions of early visual analysis such as stimulus orientation and retinal position (see Fahle, 2005). Therefore it is frequently inferred that perceptual learning occurs as a result of neural plasticity at early stages of visual analysis that are selective for these specific visual dimensions. Indeed, a number of neuro-imaging studies have presented converging evidence for an early locus of perceptual learning (e.g., Schwartz et al., 2002; Furmanski et al., 2004; Pourtois et al., 2008). Consequently, the design of Experiment 1 is typical of the approach previously used with achromatic stimuli, as evidenced in existing perceptual learning literature (see Sasaki et al., 2010; Lu et al., 2011, for reviews). As such, it provides an important bridge between studies of perceptual learning in the color domain and studies of perceptual learning more generally.

Participants were randomly allocated to one of four training conditions in which stimuli varied in location (top or bottom) and hue (green or blue). A chromatic threshold task was performed by all participants before and after a training phase. During the training phase, participants were trained at just one retinal position and hue, either ‘top green,’ ‘top blue,’ ‘bottom green’ or ‘bottom blue.’ This design enables measurement of the effect of training on chromatic thresholds at the training hue/location. Additionally, it enables the extent of generalization of learning to another location, to other hues in the same color category (‘near’ ±2.5 Munsell hue units relative to the training hue), and to hues in a different category (‘far’ 17.5, 20 or 22.5 Munsell hue unit difference from the training hue). The extent of transfer of learning across retinal locations will provide some constraints on the stage of processing at which such effects occur. At early stages of visual processing (e.g., V1), cell receptive fields at the stimulus eccentricity used in Experiment 1 tend to be small and receptive field sizes increase through later stages of visual processing (Kastner et al., 2001; Smith et al., 2001). Therefore, if chromatic perceptual learning does not transfer across locations, this might imply that learning is localized to relatively early stages of visual analysis that code for retinal location.

### Method

#### Participants

Fifteen paid volunteers (mean age 24.0 years; range 19–31) took part in the experiment.

In all experiments, all participants had normal or corrected-to-normal vision and normal color vision as assessed by the City University Color Vision Test (Fletcher, 1980). Informed written consent was obtained from all participants and the work was conducted in accordance with the guidelines of the University of Surrey research Ethics Committee.

#### Design

The experiment followed a test-training-test design. The same stimuli and procedure were used in both the training and test phases. During test phases participants’ chromatic thresholds for discriminating the orientation of an oblique chromatic boundary were measured at two different visual field positions (upper right and lower right) and for two different hue regions (blue and green). During training the participants practiced the same task over 8 days but for just one visual field position and hue region. Participants were assigned to one of four training groups formed from all the possible combinations of two hue regions and two visual field positions.

A summary of the experiment schedule can be seen in **Table 1**.

**Table 1:** Summary of the schedule for Experiment 1.

	Day 1	Days 2–9	Test day
Top green group	All stimuli threshold task	Top green threshold task	All stimuli threshold task
Top blue group	All stimuli threshold task	Top blue threshold task	All stimuli threshold task
Bottom green group	All stimuli threshold task	Bottom green threshold task	All stimuli threshold task
Bottom blue group	All stimuli threshold task	Bottom blue threshold task	All stimuli threshold task

*Participants were randomly allocated to one of four training groups (top green, top blue, bottom green, bottom blue). On day 1 and day 10 participants’ discrimination thresholds in both color regions (green and blue) were measured at both locations (top and bottom). On days 2–9, participants were trained only at their training location and on their training hue.*

#### Apparatus and Experimental Set-Up

Stimuli were displayed on a 21-inch Eizo Flexscan F980 CRT monitor (resolution 1024 × 768 pixels subtending a visual angle of 39.4° × 29.4°) controlled by a Ventrix 511 computer. Stimuli were generated by a Cambridge Research Systems (CRS; Rochester, UK) Visual Stimulus Generator (VSG) 2/5 graphics card. The look-up tables of this palette based, graphics system were manipulated to generate 15-bit per gun output resolution to produce the color stimuli. A high-resolution timer DLL (ExacTics) ensured accurate event timing. The monitor and VSG system were calibrated using CRS software in combination with a CRS ColorCal colorimeter. Participants’ responses were made using a game pad (PCL RP100) and they received auditory feedback.

Participants viewed the monitor in a darkened room at eye level to the center and from a viewing distance of 57 cm, maintained by a chin and forehead rest.

#### Stimuli

**FIGURE 1:**
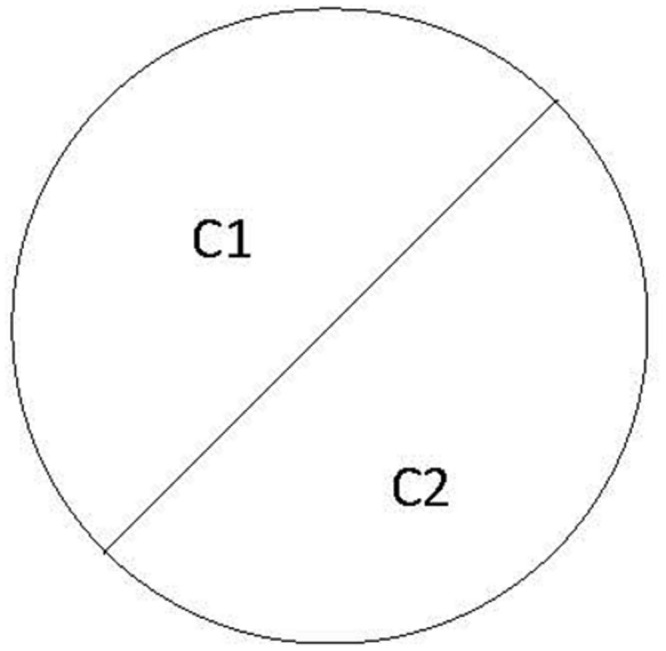
**Schematic representation of a stimulus used in Experiments 1 and 2.** Two colors (C1, C2) fill the halves of the circle, with the resultant chromatically defined edge pointing either to the left or to the right.

The stimuli consisted of two semi-circles of color abutted to make a circle of 10° diameter (see **Figure 1**) displayed against a luminance matched background (mean luminance 30.5 cd/m2).

The two halves of the circle were designed to be isoluminant. Nevertheless, due to display non-uniformity over space and time, and variation in the isoluminant point with retinal location (Schiller et al., 1991), it is possible that there may be very small residual luminance differences that could be used by observers to detect the chromatic boundary. To prevent this, on each trial, the stimuli were embedded in static, Gaussian, luminance noise, which serves to swamp any residual luminance signal that could be used to detect the boundary (Snowden, 2002). To generate the Gaussian noise each pixel across the face of the display was set to one of 250 possible grayscale values, randomly selected from a Gaussian distribution (standard deviation = 0.3) that was normalized to have a mean luminance equal to the chromatic stimulus luminance (30.5 cd/m2). A new noise mask was generated for every stimulus presentation to prevent adaptation to the noise mask.

**FIGURE 2:**
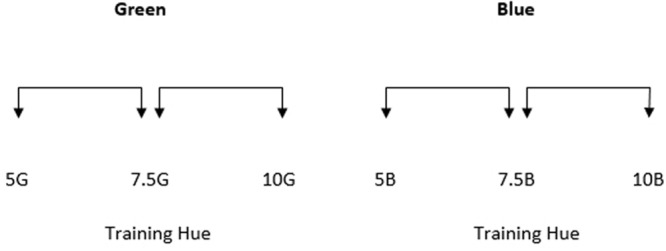
**The Munsell hue co-ordinates of the six test points used in Experiment 1.** Value and chroma were both 6. The green and blue training hues were 7.5G and 7.5B respectively. The arrows between each stimulus indicate that stimulus separation sizes are intended to be equal with test points separated by 2.5 Munsell Hue units.

The colors of the two halves of each stimulus were generated around six test points (see **Figure 2**). Three of these test points were from the green region of color space and three were from the blue region. All stimuli were generated from within the green and blue regions of color space as this is a comparatively large region of color space that enables greater flexibility in stimulus selection and has consequently been used in many studies investigating chromatic perception and color category effects. Test points were equally spaced in Munsell Hue,^1^ separated by 2.5 Munsell Hue units. Value and Chroma were kept constant at 6. The central test points in each region of color space (Munsell Hue 7.5G and 7.5B for the green and blue regions respectively) were the two training hues.

Stimuli were presented in one of two locations. For the ‘top green’ and ‘top blue’ groups, stimuli were centered 7° from fixation in the upper right quadrant of the visual field. For the ‘bottom green’ and ‘bottom blue’ groups, stimuli were centered 7° from fixation in the lower right quadrant of the visual field. The two locations were 9.9° apart (center to center).

In all experiments, the chromaticity co-ordinates of all stimuli were checked and verified with a Cambridge Research Systems ColorCal colorimeter at regular intervals during data collection. All colors were rendered as viewed under Illuminant C. All stimuli were presented with rectangular temporal profiles.

#### Procedure

During the training and test phases participants were instructed to press one of two buttons on a game pad with their right hand to signify whether the chromatic edge tilted to the left or to the right. Participants were asked to be as fast and as accurate as possible. Before the experimental session a practice session of five trials was performed using randomly selected colors from one of the six test points. Each trial was preceded by the presentation of a 0.5 cm diameter black fixation dot, which appeared centrally on a uniform gray background (mean luminance 30.5 cd/m2). The participant initiated each trial with a key press after which the black fixation dot was displayed for 249 ms, followed by the stimulus for 125 ms. In each trial, the circle was divided obliquely in half by a chromatically defined edge that tilted 45° either to the left or to the right (see **Figure 1**). This large difference in orientation (90°) was selected because such a difference is readily discriminated, loading task performance more on detection of the chromatic boundary *per se* rather than orientation discrimination. The two orientations were presented randomly across trials and there were six blocks in total, one for each test point.

Discrimination thresholds were measured using the ZEST algorithm (King-Smith et al., 1994), which varied the size of the hue difference between the two halves of the circle. The ZEST algorithm is a Bayesian adaptive threshold estimation procedure that continuously modifies an assumed *a priori* probability density function (PDF). The PDF represents the probability that threshold is at each value within a range of chromatic differences. It is calculated on the basis of the preceding responses and sets the difficulty of the next trial to be the mean of the current PDF function. In this way all of an observer’s previous responses are taken into account in setting the difficulty of the next trial. Within each block three ZEST runs lasting 32 trials were randomly interleaved and threshold was estimated as the mean of the three runs.

### Results

Statistical analysis was conducted using Analysis of Variance (ANOVA) with Bonferroni corrected *post hoc* tests. Greenhouse–Geisser adjustments to the degrees of freedom were performed where appropriate to correct for sphericity violations. The statistically significant findings are described below.

#### Transfer Across Hue

**FIGURE 3:**
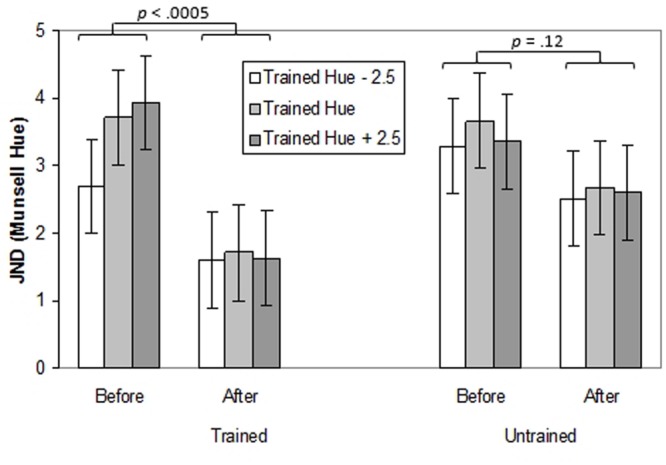
**Thresholds (in Munsell Hue units) for discriminating the orientation of a chromatically defined edge before and after training for Experiment 1.** Separate bar charts are shown for trained and untrained hues and separate bars are shown for the central ‘training hue’ and its untrained equivalent and for adjacent test points. Error bars represent ±1 SE.

A three-way ANOVA with Time (pre-training, post-training), Hue (trained hue, untrained hue) and Test Point (-2.5, 0, and +2.5 Munsell hue steps relative to the training hue) was performed. The effects of training on thresholds for the trained and untrained hues (blue or green) and on test points in the same region of color space were explored (see **Figure 3**; individual data can be seen in Supplementary Figure S1A). There was a main effect of Time indicating that generally, thresholds were lower after training, *F*(1,14) = 10.58, *p <* 0.01, $\eta_{p}^{2}$ = 0.43. In other words participants’ performance on the task improved with practice. There was also a main effect of Test Point, *F*(2,28) = 4.57, *p <* 0.05, $\eta_{p}^{2}$ = 0.25, with better performance on the -2.5 hue than the training hue (*p* < 0.05). Most importantly, there was an interaction between Time and Hue, *F*(1,14) = 4.51, *p* = 0.05, $\eta_{p}^{2}$ = 0.24. Thresholds decreased significantly for the trained hue (*p* < 0.0005) but not for the untrained hue (*p* = 0.12).

#### Transfer Across Location

**FIGURE 4:**
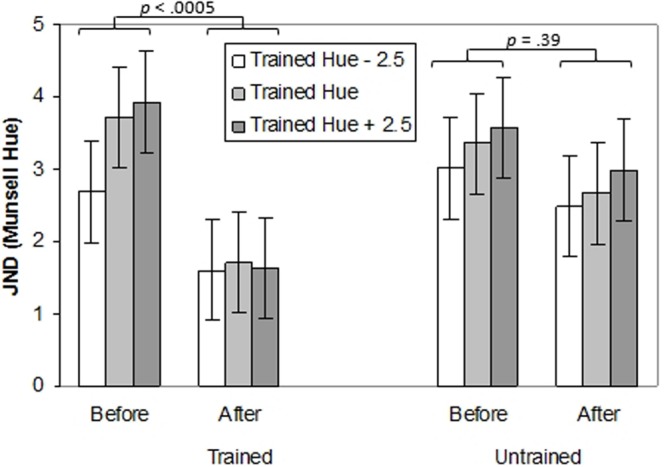
**Thresholds (in Munsell Hue units) for discriminating the orientation of a chromatically defined edge before and after training for Experiment 1.** Separate bar charts are shown for trained and untrained retinal locations and separate bars are shown for the training hue and adjacent test points. Error bars represent ±1 SE.

A three-way ANOVA with Time (pre-training, post-training), Location (trained location, untrained location) and Test Point (-2.5, 0, and +2.5 Munsell hue steps relative to the training hue) was conducted to explore the effects of training on thresholds for the trained and untrained locations (top or bottom) and on test points in the same region of color-space as the trained hue. The effects of training on thresholds for the trained and untrained locations and on test points in the same region of color-space can be seen in **Figure 4** (individual data can be seen in Supplementary Figure S1B). There was a main effect of Time, *F*(1,14) = 7.62, *p <* 0.05, $\eta_{p}^{2}$ = 0.35. indicating improvement on the task following practice. There was also a main effect of Test Point, *F*(1,14) = 5.72, *p <* 0.05, $\eta_{p}^{2}$ = 0.29. Performance was better on the test point -2.5 hue units from the trained hue than on the +2.5 test point (*p* < 0.05). Crucially, there was an interaction between Time and Location, *F*(1,14) = 7.72, *p <* 0.05, $\eta_{p}^{2}$ = 0.36. Thresholds decreased significantly at the trained location (*p* < 0.0005) but not at the untrained location (*p* = 0.39). Finally, there was an interaction between Time, Location and Test Point, *F*(2,28) = 3.35, *p <* 0.05, $\eta_{p}^{2}$ = 0.19. For the trained location but not the untrained location, before training thresholds for the -2.5 test point were significantly lower than for the trained hue or the +2.5 test point (*p’s* < 0.05), which did not differ (*p* = 1.0). After training none of the thresholds differed significantly across test points (*p’s* = 1.0).

### Discussion

Experiment 1 explored the extent to which performance on a chromatic perceptual threshold task improved with practice. The degree to which this perceptual learning transferred across and within hues was investigated. Additionally, the extent of transfer across retinal locations was analyzed to examine the potential neural loci of improvements.

The results revealed that participants had lower discrimination thresholds following 8 days of practice at making judgments about the orientation of a chromatically defined boundary. Improvements in chromatic discrimination did not transfer to a different hue category but were specific to the hue category that participants were trained on. This finding is in line with previous studies of color categorical perception (Özgen and Davies, 2002). However, improvements in chromatic discrimination did transfer across test points within a hue category. The finding that improvements in chromatic discrimination based judgments transfer within a hue category but not to a different hue category bears an intriguing resemblance to color categorical perception. As outlined previously, category effects occur when discrimination of colors that fall within a category is less accurate than discrimination of colors from different categories. Consequently, it would be plausible to expect that in the current experiment, learning would transfer equally to adjacent test points in the same color category to the trained hue, as these hues are relatively indistinct from the training hue. This was the case, with thresholds at the trained hue and test points located 2.5 Munsell steps either side of the trained hue being significantly reduced following training. However, a true test of whether transfer of perceptual learning across test points reflects the categorical structure of color space would require comparison of transfer to test points within a category and equally different test points from adjacent categories. This is explored further in Experiment 3.

It was also surprising to note that thresholds between adjacent test points within the same category differed significantly prior to training with an apparent tendency for thresholds to increase with hue value. Examination of the individual data (see Supplementary Figure S1A) shows that this pattern is not consistent across all observers, or to one particular hue. Nevertheless, previous work has shown that thresholds vary with Munsell hue within the blue–green region of color space (Roberson et al., 2009) when measured using a very similar procedure to the current experiment. Whilst the measurement points used by Roberson et al. (2009) range from 10G through to 7.5B, and therefore do not fully overlap with our present ranges of 5G–10G and 5B–10B, making a direct comparison impossible, it is interesting to note that this similar threshold variation is documented in the literature (see also Witzel and Gegenfurtner, 2013). At present we are unable to offer a fuller explanation for this variation.

Additionally, the results revealed that the performance improvement was specific to the retinal location at which participants trained and did not transfer to another location separated by 9.9° (center to center difference between the locations of the circular patches; note the edges overlapped by 0.1°). This finding sheds light on the potential neural locus of chromatic perceptual learning. At early stages of visual processing, such as V1, cell receptive fields at the eccentricity of the stimuli used in the current experiment tend to be small (0.5°). As stages of the visual processing hierarchy progress, receptive field sizes tend to increase up to around 5.5° in area V4 and to 20° or more in anterior inferotemporal cortex (Kastner et al., 2001; Smith et al., 2001). Therefore, whilst far from conclusive, the lack of transfer of perceptual learning across retinal location observed would appear to rule out learning based on higher level task strategies and is consistent with learning that is localized to relatively early, retinotopically mapped stages of visual analysis that also deal with the processing of chromatic stimulus properties. It is important to note that the neural locus of learning cannot be precisely determined from psychophysical data as spatial information is widely preserved to varying extent throughout the visual processing system. However, it is also worth noting that the inferences drawn from psychophysical approaches, such as those used here, have often been borne out by converging evidence from neuroimaging methods, which have shown cortical changes, occurring with perceptual learning, as early as V1 (e.g., Sagi, 2011).

Thus, overall, these findings suggest that chromatic perceptual learning, as assessed by a perceptual threshold task, occurs at relatively early stages of visual analysis. Experiment 2 investigates the effect of category training on chromatic thresholds. If color categorical perception effects result from chromatic perceptual learning then we should expect that learning color categories can drive changes in chromatic thresholds that result in categorical color perception.

## Experiment 2: The Effect of Category Training on Chromatic Thresholds

Experiment 2 tested whether learning novel color categories can induce changes in chromatic thresholds consistent with categorical perception of color. Thus, unlike Experiment 1, different tasks were used during the training and test phases. The training phase in Experiment 2 was identical to that used by Özgen and Davies (2002), consisting of a categorization task that was conducted over three sessions, occurring on three consecutive days (see also Kwok et al., 2011). During training, participants learned to categorize stimuli from the green region of color space into two new categories distinguished by a new color boundary. On day four participants undertook a short refresher training session, followed by the chromatic threshold task from Experiment 1 applied to the green and blue regions of color space. Participants in the control group performed the threshold task with no prior training. The adoption of a between group design, with no pre-testing phase and a shorter training duration compared to Experiment 1, produced a shorter overall experiment facilitating recruitment of a larger sample than Experiment 1.

### Method

#### Participants

A group of 49 paid volunteers were randomly allocated to training and control groups. There were 24 participants (mean age 25.1 years) in the training group and 25 participants (mean age 25.4 years) in the control group.

#### Design

Participants in the training group first completed a training phase over 3 days during which they learned to categorize stimuli from the green region of color space into two new categories. On the fourth day participants completed refresher training and then their chromatic thresholds were measured using the same threshold task as Experiment 1. The control group didn’t undertake any training but just completed the threshold task. A summary of the experiment schedule is shown in **Table 2**.

**Table 2:** Summary of the schedule for Experiment 2.

	Day 1	Day 2	Day 3	Test day
Training group	Context training	Context training	Context training	Refresher training
	+	+	+	+
	Singleton training	Singleton training	Singleton training	Threshold task
Control group	–	No training	–	Threshold task

*Participants in the training group were trained in a categorization task in three sessions over 3 days. On the test day training group participants received a refresher session, followed by the target detection task and the JND task. Participants in the control group performed the target detection and JND tasks without any of the training sessions or the refresher.*

#### Apparatus and Experimental Set-Up

The set-up for the test phase was identical to that used in Experiment 1.

For the training phase, stimuli were presented on a 21-in Sony Trinitron GDM-F520 CRT monitor (display resolution 1024 × 768 pixels) using a Dell Pentium 4 computer. Stimulus presentation was controlled with Visual Basic software. Responses were made using the keyboard. Participants were seated in a darkened laboratory, and viewed the monitor at eye level using a chin rest positioned 50 cm away from the computer screen.

#### Stimuli

For the test phase the stimuli were identical to Experiment 1 with the following differences. As outlined previously, the two halves of the circle were designed to be isoluminant. However, unlike Experiment 1, noise was not used in order to reduce total testing time. This is because the requirement to generate a new noise mask on each trial substantially increases stimulus generation time and therefore trial length. The absence of masking may have meant that there were unintended luminance cues available to participants to make the tilt discrimination. However, they were clearly not able to learn to use these, or in fact the intentional chromatic differences, as a function of the category training because there were no categorical perception effects evident in the threshold data even after the successful category training (see Results below).

**FIGURE 5:**
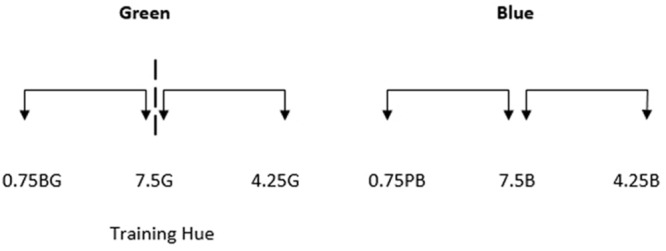
**The Munsell co-ordinates of the six test points used in Experiment 2.** Value and chroma were both kept constant at 6. The dashed line represents the new boundary within the green region of color space, which corresponds with the green training hue (7.5G). The arrows between each stimulus indicate that stimulus separation sizes are intended to be equal with test points separated by 3.25 Munsell Hue units.

Stimuli were presented in the center of the monitor. The colors comprising the two halves of each stimulus were generated around the six test points shown in **Figure 5**. As mentioned previously, the trained green boundary was at 7.5G. Participants were not trained to divide the blue region into two new categories but for consistency, the equivalent location (7.5B) is referred to as the ‘boundary.’

Test points were equally spaced in Munsell Hue, but were separated 3.25 Munsell Hue units rather than the 2.5 Munsell Hue units separations used in Experiment 1. A larger separation size between test points was selected for Experiment 2 to enable the exploration of potential category effects whilst accounting for the much shorter period of category training and the different training and test phase tasks. For all test stimuli, Value and Chroma were kept constant at 6.

**FIGURE 6:**
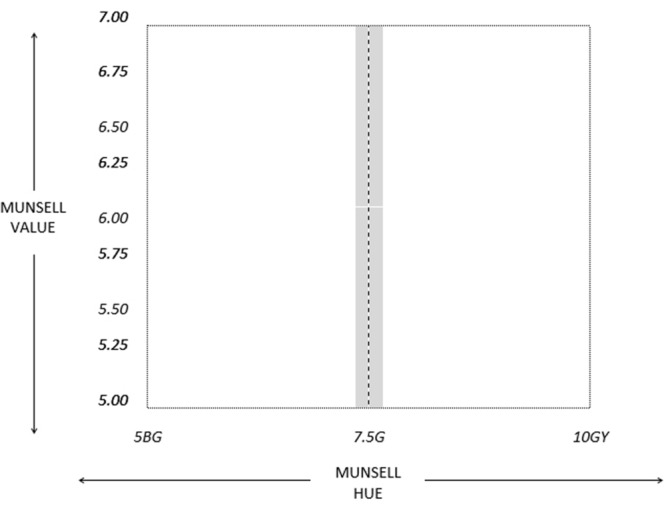
**Random color generation area from which the training stimuli in Experiments 2 and 3 were produced.** The dashed line represents the new category boundary that participants learned during training. Points closer than 0.2 Munsell Hue steps from the boundary were never used as stimuli (this excluded area is represented by the shaded region).

The stimuli used for the training phase were computer-generated colors randomly taken from an area within the green region of color space. The new hue boundary fell roughly in the center of the green linguistic category (7.5G) and stimuli were generated from the region around this. Possible Munsell Hue varied between 5BG and 10GY and Munsell Value varied between 5 and 7. Munsell Chroma was kept constant at 6. Stimuli within 0.2 Munsell Hue units of the boundary were avoided (see **Figure 6**). Stimuli were 5 cm colored squares displayed against a background of neutral gray (mean luminance 30.5 cd/m2).

#### Procedure

The procedure for the test phase was identical to Experiment 1.

**FIGURE 7:**
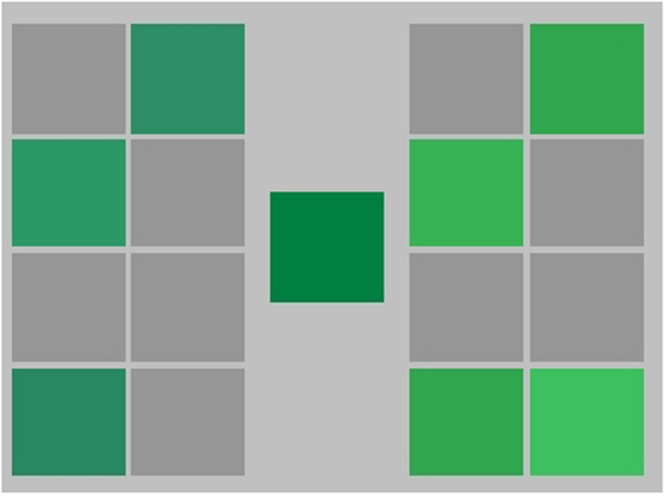
**Representation of the context training phase for Experiments 2 and 3.** Randomly generated stimuli appeared individually in the center of the screen and participants had to make a category judgment, deciding whether each stimulus belonged to the category on the right or the left of the screen.

During the training phase participants took part in three types of training: context training; singleton training; and refresher training. Context training was always completed first. For each of the context training trials, a random point within the green training area was selected and the corresponding color was displayed on the monitor. The random test color could fall on either side of the training boundary, avoiding points very close to it (see **Figure 6**). This color was presented in the center of the screen on a uniform gray background (mean luminance 30.5 cd/m2). It was flanked on either side by two grids of eight slots (two columns, four rows) to be filled with the incoming test colors (see **Figure 7**). Participants could place the first test color in any position in either grid. Once the first color was placed on the left or right side, colors from the same category had to be placed on the same side of the screen and colors from the other category had to be placed on the opposite side. Test colors remained in the center of the screen until a key press response was made. Response keys were assigned to select which grid each test color belonged to (‘left-arrow’ for category 1 or ‘right-arrow’ for category 2), and following a response immediate feedback was given. If the response was correct the color moved to the designated grid and remained in its slot, but if the response was incorrect the color moved to the designated grid and then disappeared, accompanied by a sound indicating the incorrect categorization. Participants were not given instructions on what the categorization was based on but had to learn from the feedback to complete the context training phase. When all sixteen slots were filled with correctly identified test colors a set was complete and a new one began until the criteria for successful category learning were met. As correct responses in each set increased, the number of different test colors increased, up to the maximum of 16. This first stage of training ended when a minimum of 20 sets were completed with at least three of these being error free. Participants were informed that the task would end when their performance was ‘sufficiently good.’ A typical participant completed context training in around 30 min.

After successfully completing context training participants undertook singleton training. Singleton training was identical to context training except that single test colors were presented in the center of the screen in the absence of the two grids that were present in context training. Correctly identified colors no longer remained on the screen, so that there was no visual point of reference for each categorization, unlike in context training. The criteria for completing singleton training were at least 250 complete trials including 25 consecutive correct responses. Participants were informed that the task would end when their performance was ‘sufficiently good’ and were usually able to complete it in around 10 min.

Participants in the training group undertook refresher training on the test day (day four). The procedure for the refresher phase was the same as for the training phase performed on days one to three, including both context training and singleton training. However, the refresher phase was shorter. Criteria for completing refresher context training were a minimum of 10 complete sets with at least one error free set. The criteria for completing refresher singleton training were at least 100 complete trials, with 25 consecutive correct responses. On average, context training took around 10 min and singleton training approximately 5 min on average.

### Results

#### Training Phase

All participants in the training group completed the training phase successfully. During the 3 days training phase participants completed between 500 and 1000 context training trials and between 250 and 500 singleton color trials on each day. On the test day, participants completed a refresher training session during which they performed between 150 and 250 context training trials and between 100 and 250 singleton color trials. Therefore, throughout the course of the training phase, the mean number of categorization trials completed by participants was 3300 trials. Separate statistical analyses were conducted using Analysis of Variance (ANOVA) with Bonferroni corrected *post hoc* tests to compare the percentage of incorrect responses made on each of the three training days, for the context and singleton training phases. A one-way ANOVA indicated that there was a significant difference in performance on the context training task across the 3 days [F(2,69) = 5.82, *p* < 0.005, $\eta_{p}^{2}$ = 0.22]. To investigate the pattern of performance Bonferroni corrected paired samples *t*-tests were used to compare the number of errors made on days one and two and days two and three. These revealed that there were significantly fewer errors made on day two compared to day one [*t*(23) = 2.79, *p* < 0.01, *d* = 0.56]. Performance on days two and three was not significantly different [*t*(23) = 2.47, *p* = 0.028, *d* = 0.11]. However, there was significant improvement between day one and day three [*t*(23) = 5.49, *p* < 0.001, *d* = 1.03]. A similar pattern was revealed for the singleton training task. There was a significant difference in the percentage of errors made on singleton training on each of the 3 days [*F*(2,69) = 3.48, *p* < 0.01, $\eta_{p}^{2}$ = 0.18]. Performance on the second day was significantly better than the first day [*t*(23) = 2.77, *p* = 0.001, *d* = 0.34]. However, performance on the third day, although improved, was not significantly different from day two [*t*(23) = 1.96, *p* = 0.062, *d* = 0.27]. Overall, performance improved significantly, being better on day three than on day one [*t*(23) = 3.93, *p* < 0.001, *d* = 0.64].

#### Threshold Task

**FIGURE 8:**
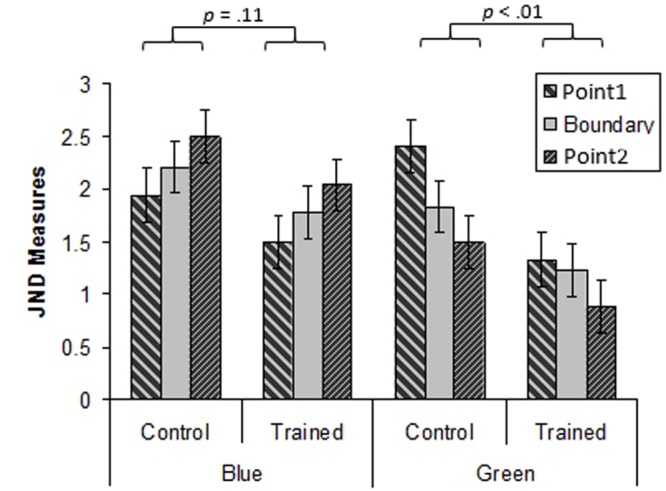
**Mean JNDs for all test points in the green and blue regions for the control and the training groups for Experiment 2.** Error bars represent subjects 95% confidence intervals.

The data can be viewed in **Figure 8**.

**FIGURE 9:**
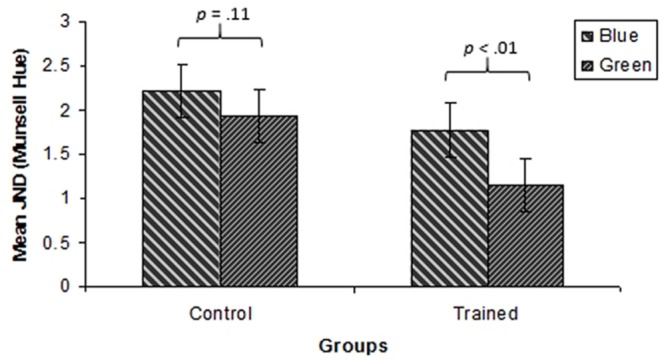
**Mean JNDs in the green and blue regions for the control and the training group for Experiment 2.** Error bars represent within-subjects 95% confidence intervals.

A three way ANOVA with Group (training, control), Hue (green, blue) and Test Point (-3.25, 0, +3.25 Munsell hue steps relative to the training hue) was performed. This showed a significant main effect of Hue, with JNDs for the blue region being higher than for the green region [*F*(1,44) = 26.6, *p* < 0.001, $\eta_{p}^{2}$ = 0.38]. The ANOVA also revealed a significant effect of Group, with the training group exhibiting lower thresholds than the control group [*F*(1,44) = 5.06, *p* < 0.05, $\eta_{p}^{2}$ = 0.10]. The interaction between Hue and Group was marginally significant [*F*(1,44) = 3.77, *p* = 0.06, $\eta_{p}^{2}$ = 0.08]. For the mean JNDs in the blue and green regions for the training and control groups see **Figure 9**. This interaction was investigated further using Bonferroni corrected paired samples *t*-tests. These revealed that JNDs were lower for the training group than the control group in the green region [*t*(44) = 2.7, *p* < 0.01], but that there was no significant difference between the groups in the blue region [*t*(44) = 1.56, *p* = 0.11]. The interaction between Hue and Test Point was significant [*F*(2, 88) = 16.87, *p* < 0.001, $\eta_{p}^{2}$ = 23] reflecting an increase in JNDs from short to long dominant wavelength in the blue region and the corresponding fall in JNDs from short to long dominant wavelength in the green region. However, there was no main effect of Test Point [*F*(2,88) = 0.13, *p* = 0.88, $\eta_{p}^{2}$ = 0.003], indicating that there was no local minimum in JNDs at the boundary when collapsed across Hues and Groups. Additionally, the interaction between Group and Test Point was not significant [*F*(2,88) = 0.78, *p* = 0.46, $\eta_{p}^{2}$ = 0.02]. Finally, the three way interaction between Hue, Group and Test Point was not significant [*F*(2,88) = 0.60, *p* = 0.55, $\eta_{p}^{2}$ = 0.01].

### Discussion

Experiment 2 sought to establish whether chromatic perceptual learning occurred on a threshold task following a period of category training on a novel hue boundary. During the 3 days training phase, training group participants learned to divide the green region of color space into two novel categories, completing an average of 3300 categorization trials. Categorization performance was found to improve on both context training and singleton training tasks. For both tasks, error decrease was greatest on day two, which suggests that category learning can occur after just one training session, consistent with the findings of Özgen and Davies (2002) and Kwok et al. (2011).

In the green region, chromatic discrimination thresholds on the threshold task were lower for the training group who had undergone training, than the control group who had done no training. However, this was not the case in the untrained blue region where there was no significant difference between the two groups. This indicates that perceptual learning was induced by the training phase and that the effects of training were evident on a chromatic threshold task. The lack of difference between the two groups in the blue region suggests that the effects of training did not transfer to the blue region. This finding is in line with the findings from Experiment 1 and with previous work suggesting that chromatic perceptual learning does not transfer to adjacent hue regions (Özgen and Davies, 2002; Clifford et al., 2012). It should be noted that by completing the training phase, training group participants had experienced a greater amount of stimulus exposure than control group participants, who had no equivalent task. However, the lack of difference between the groups in their performance on the blue color region suggests that any advantage attained by the training group as a result of their additional stimulus exposure was limited to the color region that they were trained on. Therefore perceptual learning was category specific.

However, discrimination thresholds at the new green category boundary were no different from those at adjacent test points. This indicates that there was no acquired categorical responding. Despite the apparent effectiveness of the training schedule, categorization effects from the original category structure still appear to be present, overriding any acquired category learning. These findings are not consistent with previous research showing color category effects for newly acquired color category boundaries (Özgen and Davies, 2002; Clifford et al., 2012). Even though an almost identical training procedure was used in the current study and in Özgen and Davies’ (2002) and Clifford et al.’s (2012) studies, category effects were not apparent for the training group during the threshold task. This difference in findings may be attributed to the method used to assess whether categorical perception effects were induced. It is possible that the observed category effects in Özgen and Davies’ (2002) study do not reflect purely perceptual processes and indeed, the category effects evidenced by Clifford et al. (2012) were only found in post-perceptual ERP components.

As outlined previously, the induced category effects in Özgen and Davies’ (2002) study were elicited on a successive same-different task, which involved the use of memory to distinguish between successive stimuli and so it is possible that category effects arose from memory processes rather than perceptual ones. Additionally, it is plausible that participants used labeling strategies during Özgen and Davies’ (2002) same-different task to facilitate category discrimination. The current threshold task was designed to provide a purer measure of perceptual ability separate from effects of memory and stimulus labeling, and this may be why a boundary dip was not observed. As argued by Roberson et al. (2009), the absence of the activation of verbal codes during threshold tasks could explain the absence of category effects. To explore this possibility, in Experiment 3 we use the same training method that was used in Experiment 2 but now we measure the effect of category training on a supra-threshold target detection task that has previously been shown to be sensitive to linguistic influences on categorical responding (Drivonikou et al., 2007). The task is lateralized to enable investigation of potential hemispheric asymmetries.

## Experiment 3: Hemispheric Asymmetries in Learned Color Categorical Perception

Recent work that explores whether categorical perception is equivalent in the left and right visual fields finds that color category effects are stronger in the right visual field (Gilbert et al., 2006; Drivonikou et al., 2007). Drivonikou et al. (2007) found that detecting the location of a colored target on a differently colored background was faster when the target and background were categorically different (e.g., green 1 on blue 1) than when they were just physically different (blue 1 on blue 2). However, this categorical effect was substantially more pronounced for LH processed RVF targets than for right hemisphere (RH) processed Left Visual Field (LVF) targets. Initial visual processing of the right visual field (RVF) is carried out by the left hemisphere (LH) of the brain (Hellige, 1993; Knecht et al., 2000; Toga and Thompson, 2003), which is where the language centers typically reside. It has therefore been suggested that lateralized color category effects are due to the dynamic, online influence of language on the visual analysis of color (Gilbert et al., 2006).

Evidence that LH dominance for color category effects does not arise until color terms are learned supports this speculation. Color category effects in infants and toddlers are lateralized to the RH, and LH categorical responding appears to begin around the time that the relevant color terms are taught (Franklin et al., 2008a,b). Learning color terms may highlight similarities among colors given the same term and emphasize differences among colors given different terms, leading to same-category compression and different-category expansion of perceptual color space, particularly for RVF (LH) stimuli (see e.g., Goldstone, 1994; Özgen and Davies, 2002; Clifford et al., 2012; Little et al., 2012).

Therefore Experiment 3 investigates whether learned categorical perception effects are present on a lateralized target detection task. Participants in the training group learned to divide green into two new categories using the same method as Experiment 2. The effects of learning were then assessed using a target detection task (Drivonikou et al., 2007), which compared discriminations that straddled the newly learned boundary with same-category discriminations either side of the boundary. Participants in the control group performed the target detection task with no prior training. If category training induces category effects, the training group should show peak discrimination around the new boundary, whilst the control group should show poorest discrimination for this region. To confirm that any difference between the control and training groups was due to category training, both groups also performed the target detection task for equivalent points in the blue region of color space with no prior category training. If observed differences between the groups are due to training, then there should be no differences in the performance of the two groups on stimuli from the blue region of color space. If left hemisphere lateralized category effects are found following categorization training, this would provide converging evidence for the role of language in color categorical responding and shed light on the processes involved in category learning.

### Method

#### Participants

Forty-six paid volunteers were randomly allocated to the training and control groups. The training group consisted of 21 participants (mean age 23.6 years) and the control group consisted of 25 participants (mean age 24.2 years). Only participants in the training group completed the training phase. The target-detection task used for the test phase was performed by all participants in the training and control groups.

#### Design

Participants in the training group undertook the same 3-day training phase as outlined in Experiment 2. On the fourth day participants completed refresher training followed by a test phase, which consisted of a target-detection task. The control group didn’t undertake any training but just completed the target-detection task.

#### Apparatus and Experimental Set-Up

The set-up was identical to the training phase of Experiment 2 except that responses for the target detection task were made using a game-pad in order to ensure high accuracy response time (RT) measurement.

#### Stimuli

The stimuli used in the training phase were identical to those used in the training phase of Experiment 2.

During the test phase, stimuli were presented as a single circular target 30 mm in diameter. The target appeared on a chromatically different, uniform background with a visual angle of 3.5°. The target could appear in one of 12 equally separated (30°) locations (see also Drivonikou et al., 2007) on a notional circle of 110 mm diameter around the fixation cross at the center of the monitor (∼12.5° from fixation). Six locations were in the RVF and six were in the LVF.

**FIGURE 10:**
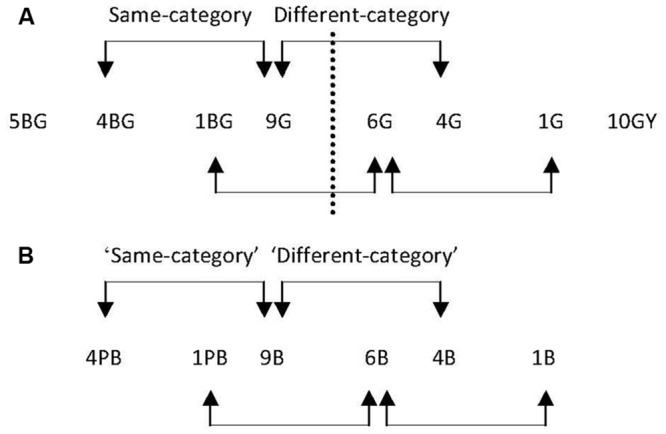
**(A)** The pairs of stimuli in the green region of color space in Experiment 3. The dashed line represents the new boundary (7.5G): there are two same-category pairs, and two different-category pairs, indicated by the arrows. The range of the trained region was from 5BG to 10GY. **(B)** The pairs of stimuli in the blue color region. No boundary is indicated as neither training nor control participants were trained to divide the blue region into two new categories.

There were two same- and two different-category pairs for the green region (see **Figure 10A**) and two ‘same-’ and two ‘different-category’ pairs for the equivalent blue region (see **Figure 10B**). Although category training was only conducted in the green region, for consistency and ease of reference, the equivalent pairs in the blue region are called ‘same-’ and ‘different-category’ pairs. Target-background separations were 5 Munsell Hue steps, with Value and Chroma constant at 6 and 6 respectively.

#### Procedure

The procedure for the training phase was identical to the training phase of Experiment 2.

On each trial of the target-detection task, trial sequences began with a 1000 ms presentation of a white fixation cross on a black background, followed by a test display where the target appeared for 250 ms and the colored background remained present until a response was made. This longer presentation time (in comparison to 125 ms for the threshold task in Experiments 1 and 2) was chosen in order to mirror previous work (Drivonikou et al., 2007) exploring category effects using lateralized target detection tasks and to allow time for linguistic influences to manifest (see Rugg and Coles, 1995). The task was to decide whether the target appeared to the left or right of fixation. Responses were made on a game pad with the left index finger indicating left, and the right index finger indicating right. Reaction times were measured from the onset of the target display until a response was made. No feedback was given during either practice or experimental trials.

There were a total of 336 trials. For each color region (blue or green) there were 168 trials made up from 42 trials for each condition: same-category left; same-category right; different-category left; and different-category right. Each stimulus in a same or different category pair served for half the trials as the target and half as the background. For each pair, the target appeared on the left for half the trials and on the right for half the trials in randomized order. Within the latter constraint, target locations were chosen at random, but with the overriding constraint that each location was used equally often across each set of 42 trials. Conditions were presented in a random order for each participant. The green and blue regions were tested in separate blocks and the order of blocks was counterbalanced across participants.

### Results

**FIGURE 11:**
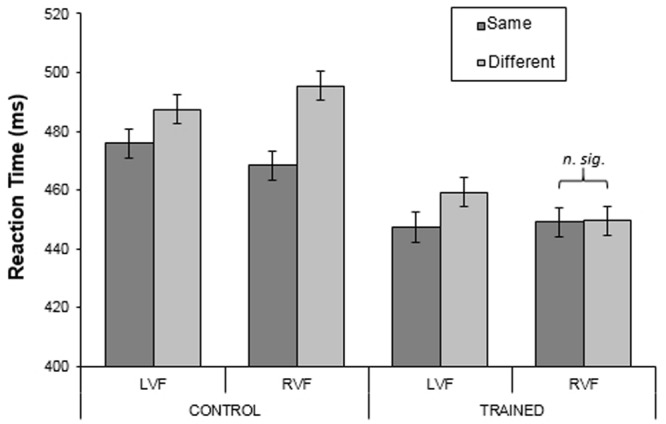
**Mean reaction times for the control and training groups in the green region for Experiment 3.** Only training participants had received training on the green region. Error bars represent 95% within-subject confidence intervals calculated by using the error term from the three-way interaction.

**FIGURE 12:**
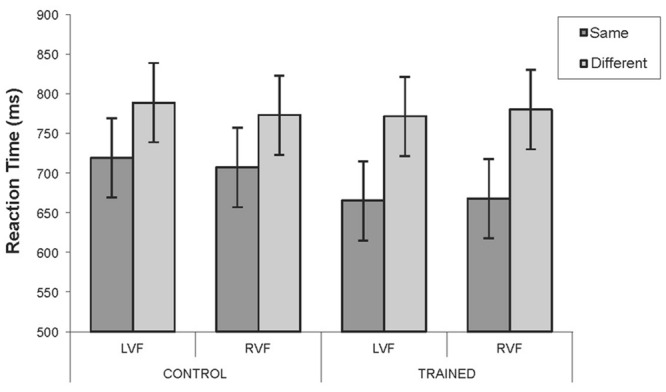
**Mean reaction times for the control and training groups in the untrained blue region for Experiment 3.** Blue region: Mean RTs for target detection for the control and the training group for each combination of visual field and category. Error bars represent 95% within-subject confidence intervals calculated by using the error term from the three-way interaction.

An initial four-way ANOVA was conducted: Color Region (blue/green) by Category (same/different) by Visual Field (LVF/RVF) by Group (training/control), with the first three factors being repeated measures. The four-way interaction was not significant. However, an inspection of the error rates showed that there were far more errors made in the blue region (∼30%) than in the green region (∼2%) by both groups. Thus, the mean RT’s for the blue region are based on substantially fewer trials resulting in a less precise estimate of the mean. This can be seen by comparing the much larger confidence intervals in **Figure 12** (blue) compared to **Figure 11** (green). Therefore to reduce the chance of a false negative error, given previous work has reported visual field specific effects (Gilbert et al., 2006; Drivonikou et al., 2007), the data for the two regions were analyzed separately. Analysis of the green region is reported first followed by the analysis of the blue region. The initial analyses were three-way ANOVAs: Category (same/different) by Visual Field (LVF/RVF) by Group (training/control), with the first two factors being repeated measures. Separate follow-up two-way ANOVAs (category by field) for each group were conducted if the three-way interactions were significant, and are reported under separate sub-headings.

#### Green Region

The percentage of correct responses was calculated for each participant, for each combination of category and visual field. The means across subjects were very similar for the two groups (97.81 and 98.83% for the control and training groups respectively). There were no significant main effects or interactions (largest *F* = 2.44; smallest *p* = 0.13). For each subject, median RTs for correct trials were calculated for each combination of category (same/different) and visual field (LVF/RVF) for each observer. **Figure 11** shows the mean RTs across subjects (mean of the subject’s median RTs) for each group. Analysis showed that there was a significant effect of Category [*F*(1,44) = 11.22, *p* < 0.01, $\eta_{p}^{2}$ = 0.20]; same-category responses were ∼13 ms faster than different-category responses. In addition, there was a strong three-way interaction between Visual Field, Category and Group [*F*(1,44) = 14.22, *p* < 0.001, $\eta_{p}^{2}$ = 0.24]. From **Figure 11**, this appears to be due to the category effect for the training group in the RVF differing from all the other group-by-visual-field combinations. Specifically, in all combinations, there appears to be a ‘reverse-category effect’ (‘same-’ < ‘different-category’), whereas for the training group in the RVF same- and different-category conditions were virtually identical.

#### Control Group

Analyzing the groups separately showed that for the control group, RTs in the two visual fields did not differ [*F*(1,24) = 0.002, *p* = 0.97, $\eta_{p}^{2}$ = 0.00]. However, ‘same-category’ targets were identified more quickly than ‘different-category’ targets (means: 472 and 491 ms for same- and different-category respectively; *F*(1,24) = 12.75, *p* < 0.01, $\eta_{p}^{2}$ = 0.35). In addition, the interaction between Category and Visual Field was significant [*F*(1,24) = 8.91, *p* < 0.01, $\eta_{p}^{2}$ = 0.27]. As can be seen in **Figure 11**, this appears to be due to the larger difference between ‘same-’ and ‘different-category’ targets for the RVF than for the LVF: this difference was on average 17 ms larger in the RVF than in the LVF [*t*(24) = 2.99, *p* < 0.01].

#### Training Group

For the training group, there was no main effect of Visual Field [*F*(1,20) = 0.57, *p* = 0.46, $\eta_{p}^{2}$ = 0.03] and overall the same-and different-category RTs did not differ [*F*(1,20) = 1.38, *p* = 0.25, $\eta_{p}^{2}$ = 0.07]. However, there was a two-way Category by Visual Field interaction [*F*(1,20) = 5.81, *p* < 0.025, $\eta_{p}^{2}$ = 0.23]. As **Figure 11** shows, the interaction reflects faster identification of same-category targets than different-category targets in the LVF [*t*(20) = 2.20, *p* < 0.05], but no such effect for the RVF [*t*(20) = 0.39, *p* = 0.70]. Therefore, training reversed the effects shown by the control group.

#### Blue Region

The percentage of correct responses was calculated for each combination of Visual Field (LVF/RVF), Category (same/different) and Group (training/control). The percentage of correct responses for the control group was 66.38%, and for the training group was 72.90%. In addition, participants were more accurate on same- than different-category trials [*F*(1,44) = 16.58, *p* < 0.001, $\eta_{p}^{2}$ = 0.36]; 73.09 versus 66.19% respectively. All other main effects and interactions were not significant (largest *F* = 1.19; smallest *p* = 0.28).

**Figure 12** shows the mean RTs for each combination of Visual Field (LVF/RVF), Category (same/different), and group (training/control). Overall, the training group responded similarly to the control group (means: 721 and 746 ms respectively). However, same-category responses tended to be faster than different-category responses [689 and 778 ms respectively; *F*(1,44) = 9.03, *p* < 0.005, $\eta_{p}^{2}$ = 0.17]. No other main effects or interactions approached significance (largest *F* = 0.78; smallest *p* = 0.36). To check that the similar RTs for the two groups were not due to different speed-error trade off functions for the two groups, the above ANOVA was repeated with accuracy as a covariate. The analysis revealed essentially the same pattern with Category being the only significant effect [*F*(1,43) = 9.03, *p* < 0.005, $\eta_{p}^{2}$ = 0.16]. Same-category trials were still about 90 ms faster than different-category trials. All other main effects and interactions remained non-significant (largest *F* = 1.36; smallest *p* = 0.251).

### Discussion

The present findings suggest that the observation of categorical perception following category learning may be task dependent. Exactly the same training was undertaken in Experiments 2 and 3 but in Experiment 2 chromatic thresholds were used to probe for evidence of category effects whereas in the present experiment a lateralized supra-threshold target detection task was used. Target detection tasks are appropriate for examining the mechanisms underlying categorical responding as there is no reliance on memory. Additionally, they also enable exploration of whether any potential category effects vary with the visual field that the stimuli are presented in. Whereas no category effects were found for the threshold task in Experiment 2, in the present target detection task there was some indication that category effects were beginning to emerge around the newly learned category boundary but only in the RVF, which is initially left hemisphere processed. An important caution is that this interpretation is based on the absence of a difference between same and different category trials in the RVF in contrast to the faster responding seen for same category trials in the LVF and for the untrained blue region.

Since Gilbert et al.’s original finding, lateralized color category effects have been replicated for speakers of other color lexicons (e.g., Roberson et al., 2008), for ERP measures (e.g., Liu et al., 2009; Mo et al., 2011), in a functional MRI study (Siok et al., 2009), and in one other study of color category learning (Zhou et al., 2010). Lateralized color category effects have also been found in the categorization of a range of different stimuli, such as cats and dogs (Gilbert et al., 2008), orientation stimuli (Franklin et al., 2010), and newly learnt form categories (Holmes and Wolff, 2012). However, the study by Holmes and Wolff (2012) showed that RVF lateralized category effects can be induced even when verbal labels for the novel categories do not appear to have been learnt. This finding challenges the explanation that LH lateralized category effects are due to the involvement of language. Additionally, a series of studies have failed to replicate a RVF lateralized category effect for color using very similar versions of the original task (e.g., Brown et al., 2009; Witzel and Gegenfurtner, 2011; Cropper et al., 2013; Suegami et al., 2014). For example, Suegami et al. (2014) used a color identification task where no spatial judgment was required and failed to find RVF lateralized category effects.

The present findings provide further evidence that categorical responding may sometimes only manifest when the online influence of language is present. Alternatively, as argued by Suegami et al. (2014), it may be that tasks involving target detection produce LH lateralized category effects due to the spatial judgment required (i.e., whether the target appears to the right or to the left of fixation). Indeed, spatial relation processing seems to be more effective in the LH (e.g., Kosslyn et al., 1989; Hellige et al., 2010), which could account for these lateralized effects on a task of this nature. However, the presence of acquired category effects on a supra-threshold task in comparison to the absence of category effects on a low level perceptual task (as evidenced in Experiment 2) following an identical period of training, suggests a great deal about the mechanisms involved in chromatic perceptual learning. It is likely that the longer stimulus presentation time (250 ms as opposed to 125 ms for Experiments 1 and 2) contributed to this finding. Stimulus duration can affect stimulus visibility (see e.g., Chaparro et al., 1993; Cropper and Derrington, 1994). Further, ERP data broadly suggests that on visual tasks of this nature, post-perceptual processes that relate to linguistic strategies occur from 210 ms onward, whereas at 125 ms in the ERP time course, early perceptual and sensory processes are indexed (see Rugg and Coles, 1995). This supports the notion that the online use of language is facilitating these effects.

It should be noted that due to the nature of the task, the point of adaptation in Experiment 3 differed from Experiments 1 and 2. This was necessary to enable replication of the task used by Drivonikou et al. (2007), for which the exact white-point cannot be unequivocally determined. During the task, participants adapted to the black background upon which the fixation cross was displayed at the start of each trial sequence. As the test display appeared for only 250 ms, there may not have been enough time to readapt. Additionally, trials consisted of different colored backgrounds and so it is possible that the adaptation related to each individual trial, or to the average of all the trials across the experiment (see Witzel and Gegenfurtner, 2011). As color appearance is always relative to the context of the respective colors and depends on the adaptation point (Fairchild, 1998), the difference in adaptation points across Experiments may have had an impact on the findings.

A prominent feature of the results was that in the untrained regions (green for the control participants and blue for the training and control participants), target detection was faster for ‘same-category’ targets compared to ‘different-category’ targets. It should be noted again that the terms ‘same-’ and ‘different-category’ are used for ease of comparison, as all stimuli within the untrained regions were from the same category. This finding could be explained in several ways. Firstly, it is possible that there were inequalities in the spacing between ‘same-’ and ‘different-category’ stimuli despite established claims that Munsell is a perceptually uniform color space. Additionally, as outlined by Hendrickson et al. (2012), the location of stimuli could play a role in this finding. ‘Different-category’ stimuli were located toward the center of the pre-existing green and blue categories, near to the location of the category prototypes, whereas ‘same-category’ stimuli were located toward the edge of the existing category structure. Discrimination has been shown to be faster and more accurate in boundary regions of perceptual categories compared to focal regions (e.g., Harnad, 1987; Kuhl, 1991; Lively and Pisoni, 1997; Özgen and Davies, 2002; Hanley and Roberson, 2011). The matched pattern of findings in the blue region for the training and control groups indicates that the partially induced category effects resulted from category learning rather than some anomalous, pre-existing difference between the two groups.

In addition, unexpectedly, target detection for blue colors was harder than for green colors. Even though the test colors were chosen so that the target-background differences in the two regions were the same number of Munsell Hue steps. One reason for poor performance for blue pairs is that detection time, as measured by RT, is substantially slower for prototypical blues than for other colors (McKeefry et al., 2003). Detecting a blue target relies almost entirely on the short-wavelength retinal cone (S cone), and the responses of the S cone appear to be slower than those of the medium- and long-wavelength cones (see e.g., Bompas and Sumner, 2008). It appears that the 250 ms stimulus presentation time was not long enough for reliable detection of a blue target.

## General Discussion

The observation of chromatic perceptual learning provides support for the possibility that color categorical perception effects arise as a result of learning during everyday experience. Specifically, the act of attending to a color category boundary may drive a learning process that enhances discrimination of colors that straddle that boundary. However, the present findings make clear that changes in chromatic thresholds *per se* are not sufficient to explain color category effects. Whilst both threshold training and learning new color categories can result in changes to chromatic thresholds, category effects may only become apparent when linguistic influences become available to mediate task performance, as suggested by Experiment 3. These language driven effects need not involve permanent modifications to the early neural circuits processing color. Instead they may be implemented dynamically as a chromatic task is performed, consistent with recent neuro-imaging evidence (Siok et al., 2009). Additionally, the current findings provide support for previous ERP research (Clifford et al., 2012), indicating that acquired color category effects are mediated by cognitive rather than perceptual mechanisms on a task of this nature. However, it is clear that before firm conclusions can be drawn there is a need to build on the present findings to explore whether extended category training leads to the development of more pronounced lateralized category effects around new category boundaries than the mere acquired absence of a ‘reverse category effect’ seen in Experiment 3. Further work could also combine neuroimaging and TMS approaches to explore the impact of disruptions to language processing on threshold and lateralized target detection tasks following category learning.

Overall, the current studies reveal that chromatic discrimination judgments improve with practice and that this improvement reflects changes at early stages of visual analysis. It is possible that this type of chromatic perceptual learning underpins color categorical perception and that category effects only appear to manifest when dynamic online influences of higher cognitive processes (such as linguistic coding) become available. These influences may underlie the differences in color category effects observed between the speakers of languages with differing color vocabularies (e.g., Roberson et al., 2000; Alvarado and Jameson, 2002; Jameson and Alvarado, 2003; Winawer et al., 2007; Roberson et al., 2009).

## Author Contributions

AG, PS, IA, VD, and ID designed the research. LN, IA, VD, and AG collected the data. PS and VD analyzed the data. AG, PS, and VD drafted the manuscript for submission. All authors contributed critical review. AG and PS revised the manuscript for publication.

## Conflict of Interest Statement

The authors declare that the research was conducted in the absence of any commercial or financial relationships that could be construed as a potential conflict of interest.

## Footnotes

1 Munsell is a standardized color metric that is based on an extensive series of psychophysical judgments (Newhall et al., 1943). It is a common color metric for equating colors in studies involving chromatic judgments (e.g., Bornstein and Korda, 1984; Gilbert et al., 2006). Munsell color space has three dimensions: Hue, Value (lightness) and Chroma (colorfulness, or saturation). A notation that consists of three co-ordinates is used to specify stimuli and each co-ordinate relates to one of the three dimensions.
