# Supplementary material for: Chromatic Perceptual Learning but No Category Effects without Linguistic Input
Source: Front Psychol. 2016 May 25;7:731. doi: 10.3389/fpsyg.2016.00731 (PMC4879779; doi:10.3389/fpsyg.2016.00731)
Supplement: Supplementary file 1 [file Data_Sheet_1.DOCX]

***Supplementary Material***

**Chromatic Perceptual Learning and Category Effects**

**Alexandra Grandison, Paul T. Sowden*, Vicky G. Drivonikou, Leslie A. Notman, Iona Alexander and Ian R.L. Davies**

***Correspondence:** Paul T. Sowden: p.sowden@surrey.ac.uk

**Supplementary Figure 1a.** Experiment 1 Individual data: transfer across hue

Observer LA – trained hue green


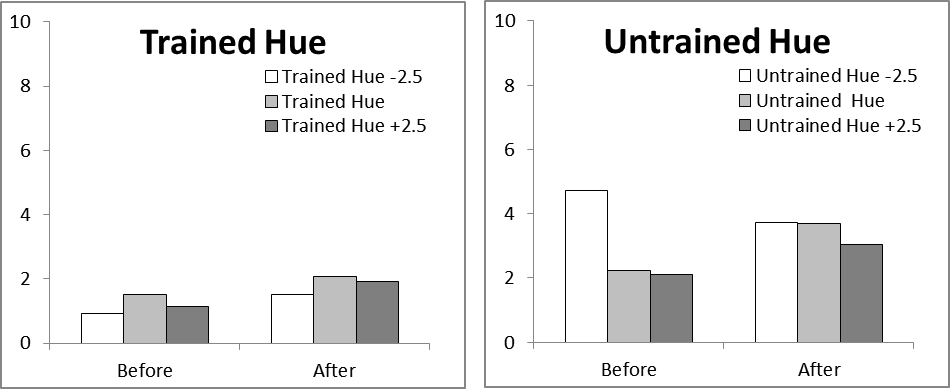


Observer K – trained hue green


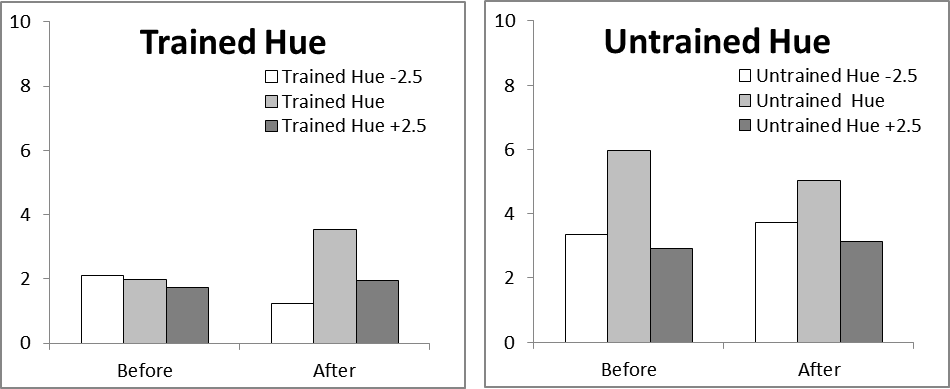


Observer SA – trained hue blue


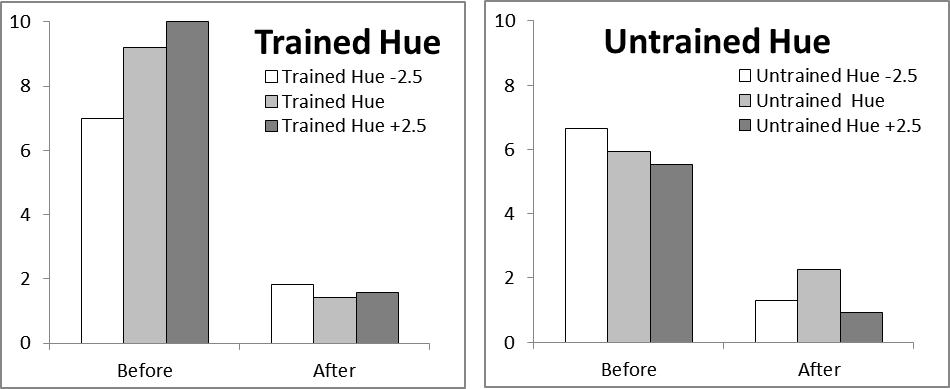


Observer AA – trained hue blue


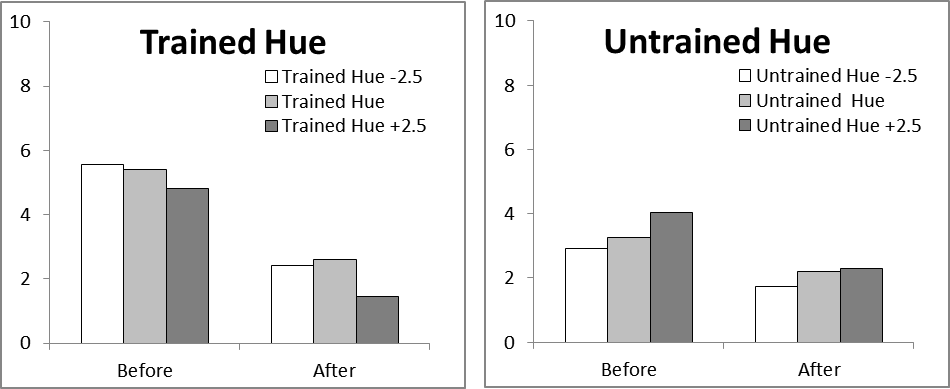


Observer SS – trained hue green


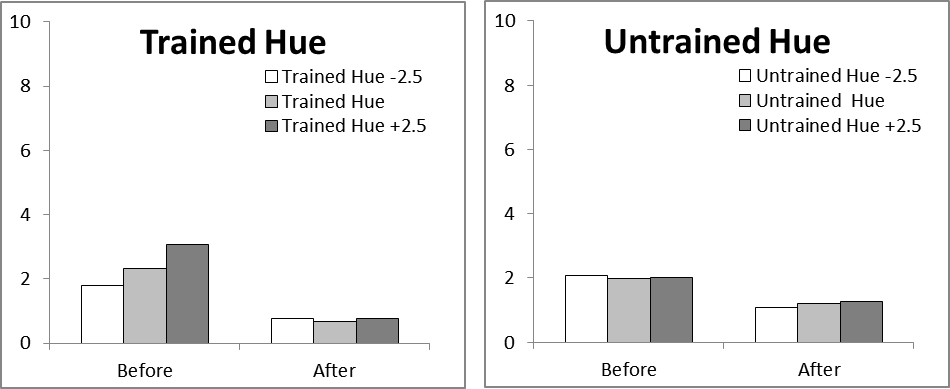


Observer CA – trained hue green


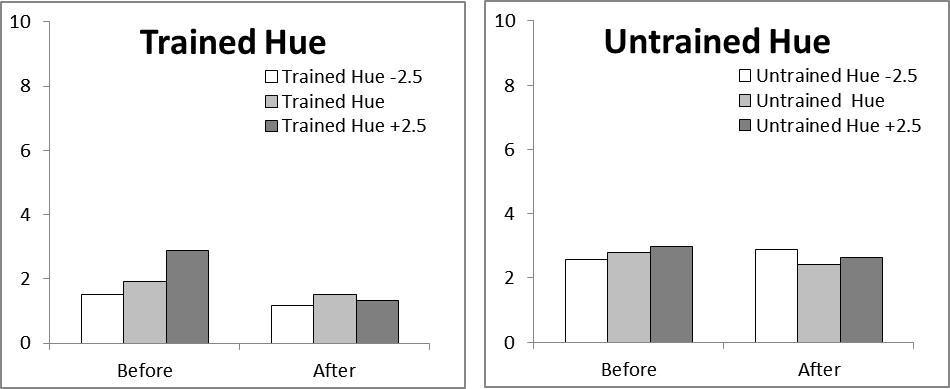


Observer EA – trained hue blue


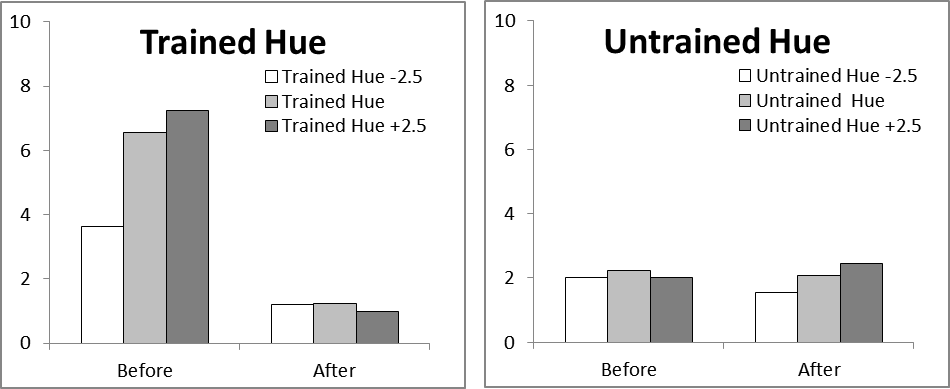


Observer H – trained hue green


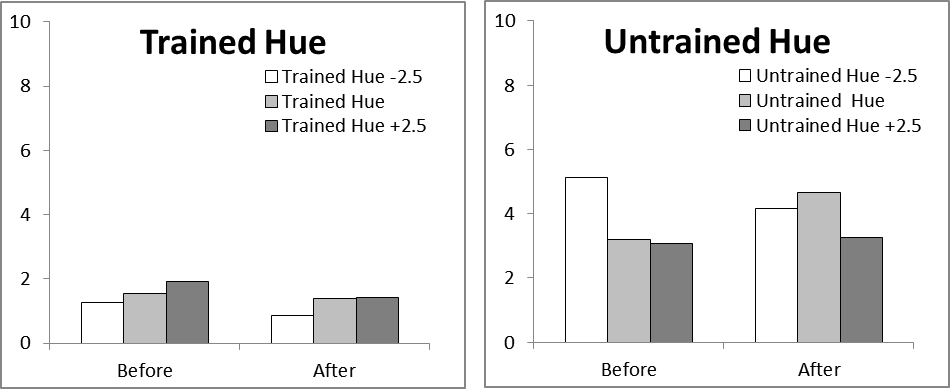


Observer AB – trained hue green


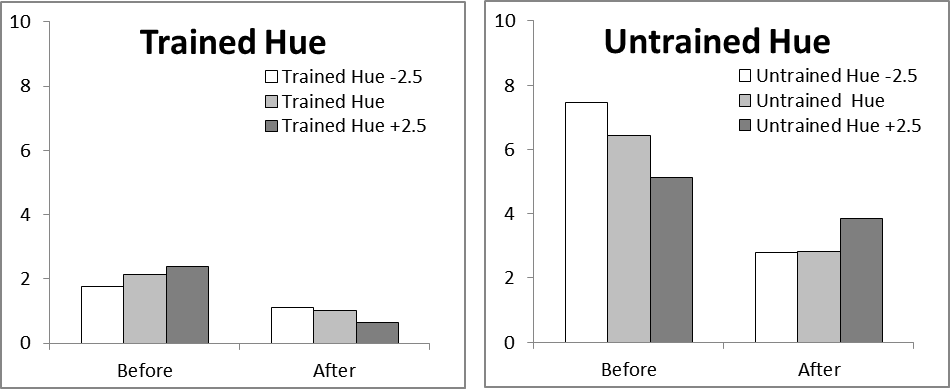


Observer S – trained hue green


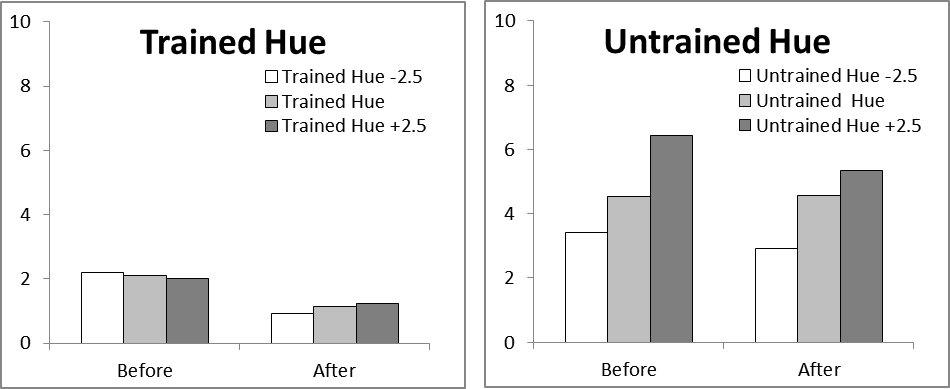


Observer JB – trained hue blue


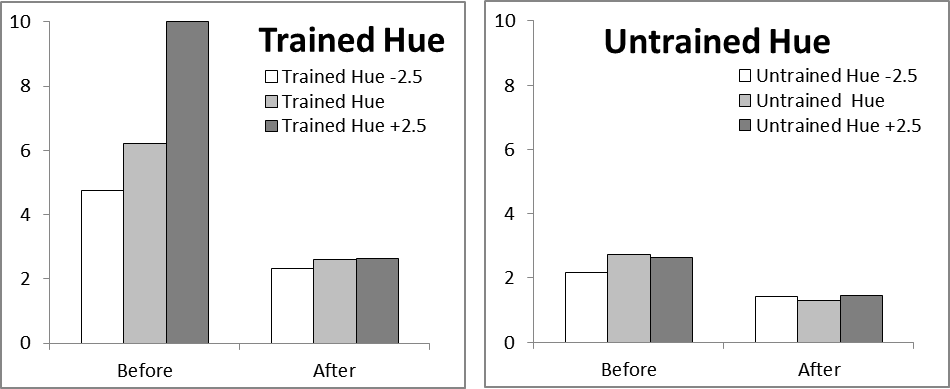


Observer EB – trained hue green


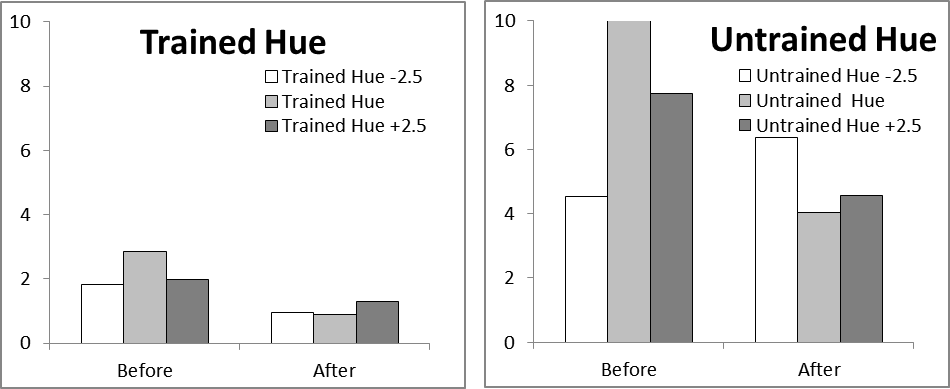


Observer SM – trained hue blue


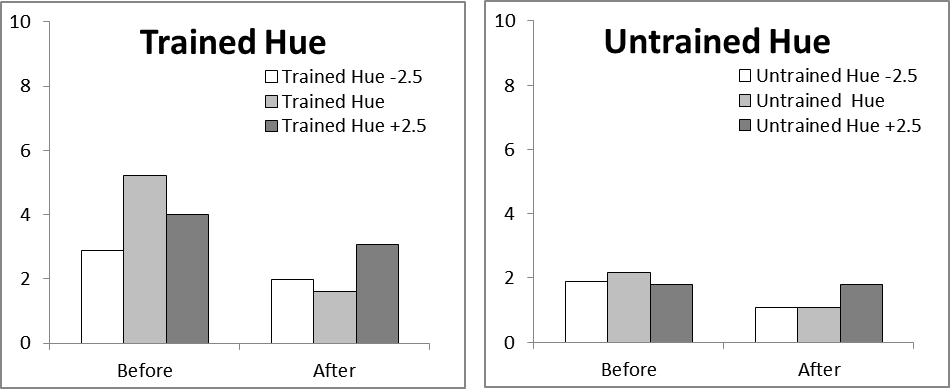


Observer LB – trained hue blue


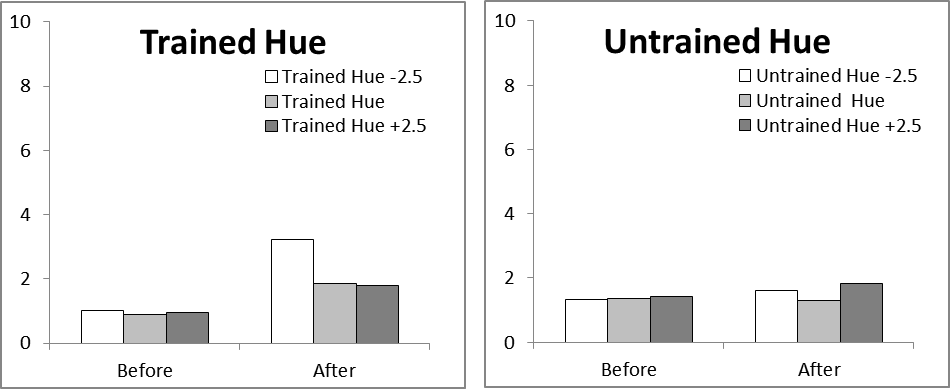


Observer CB – trained hue blue


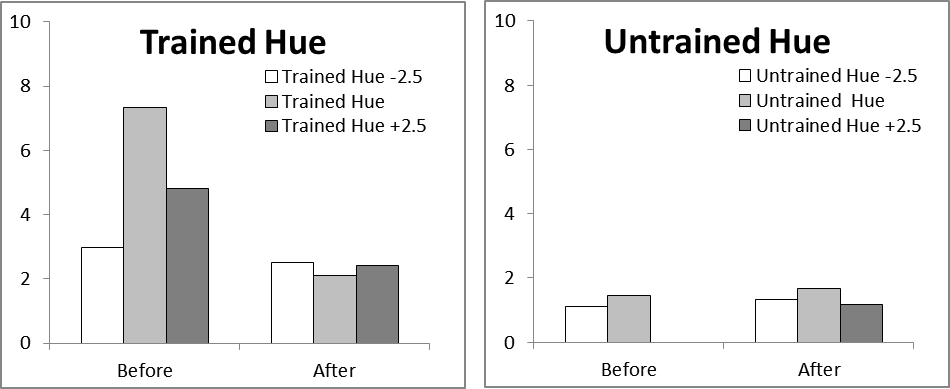


**Supplementary Figure 1b.** Experiment 1 Individual data: transfer across location

Observer LA – trained location top


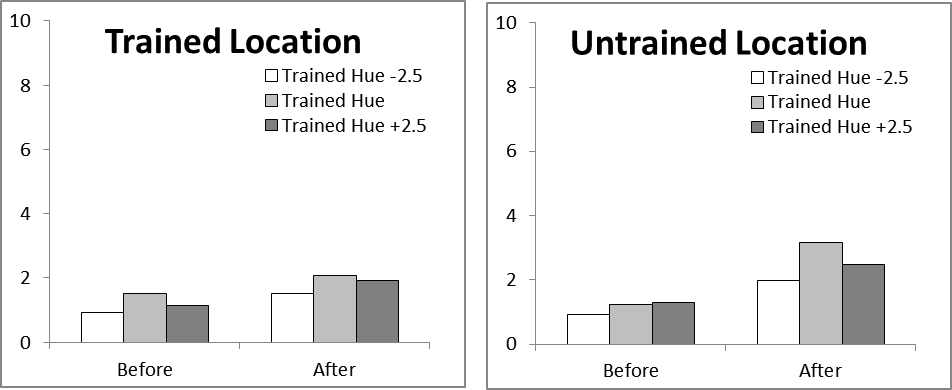


Observer K – trained location bottom


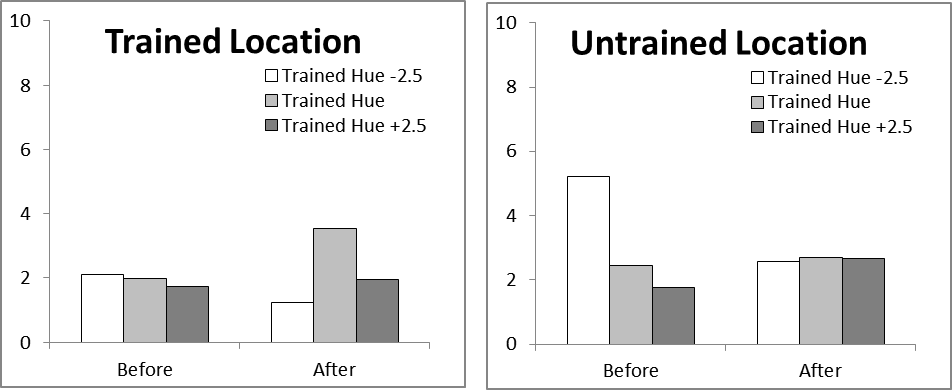


Observer SA – trained location top


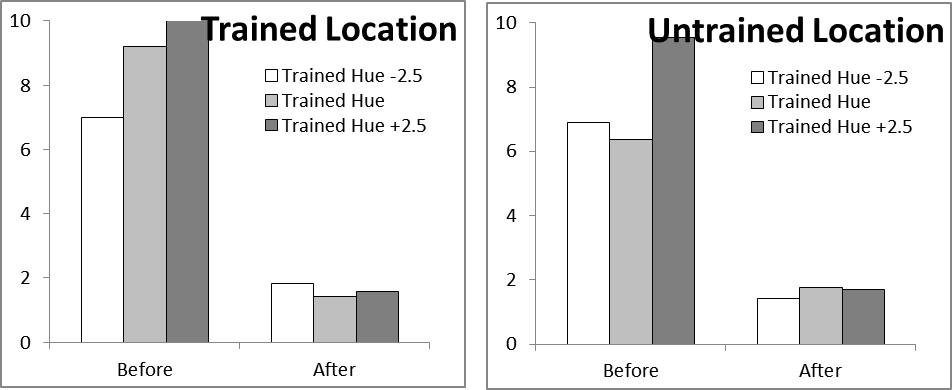


Observer AA – trained location bottom


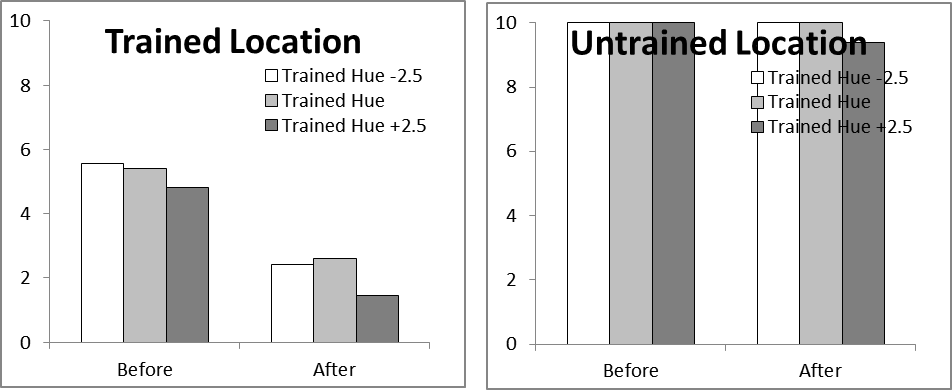


Observer SS – trained location top


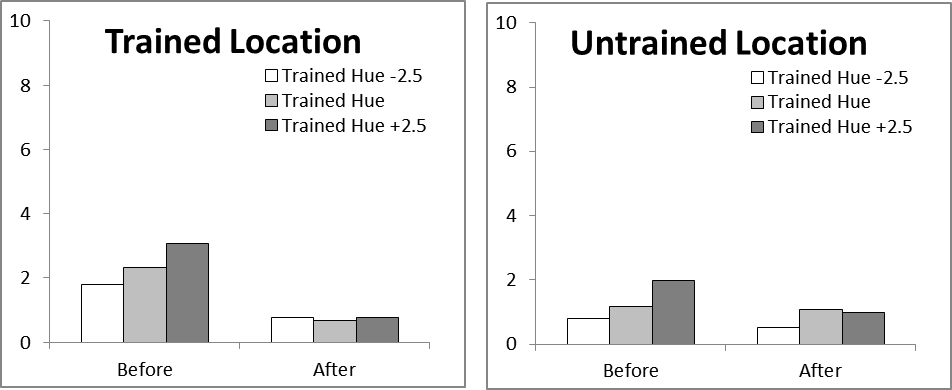


Observer CA – trained location bottom


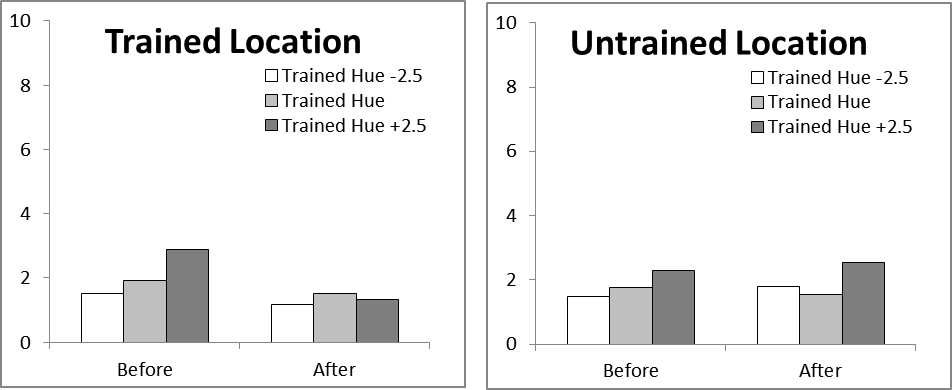


Observer EA – trained location top


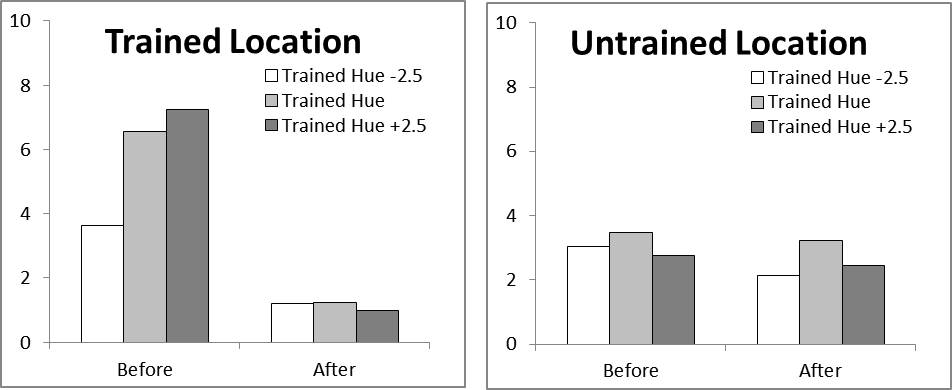


Observer H – trained location top


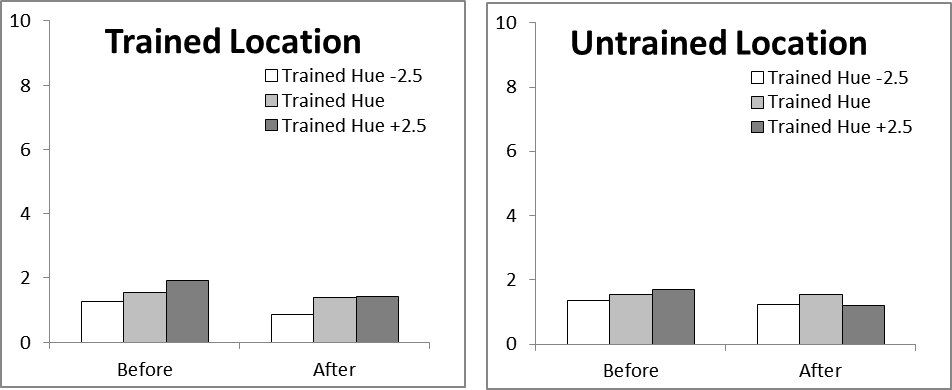


Observer AB – trained location bottom


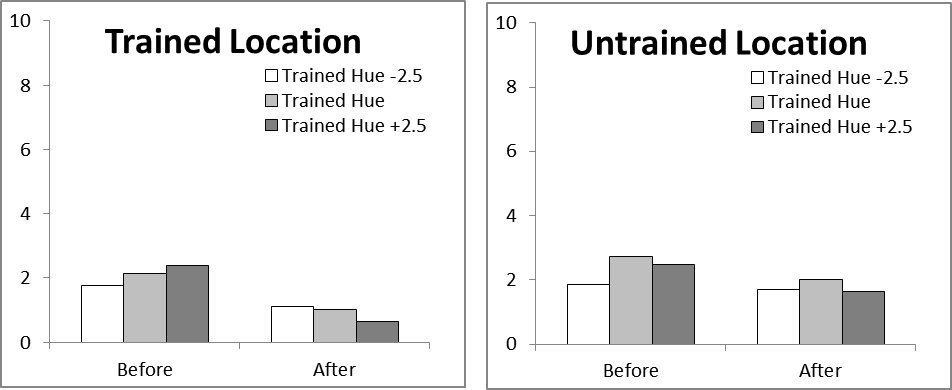


Observer S – trained location bottom


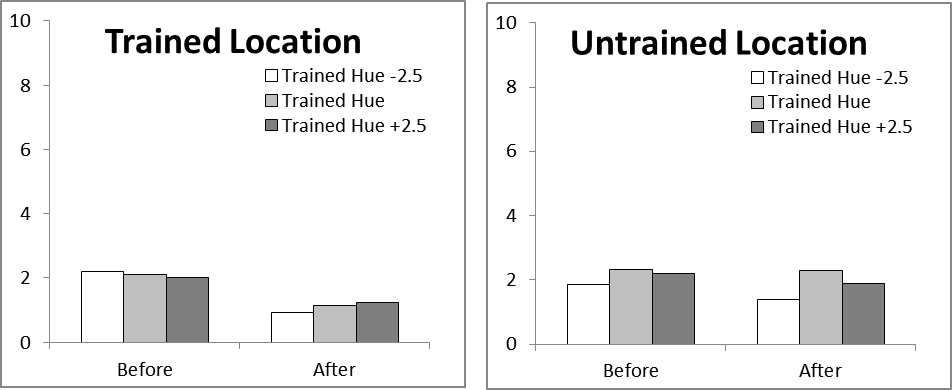


Observer JB – trained location top


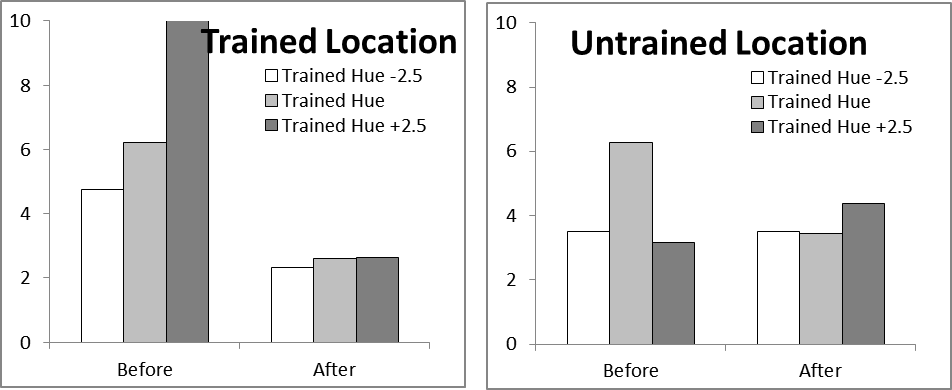


Observer EB – trained location top


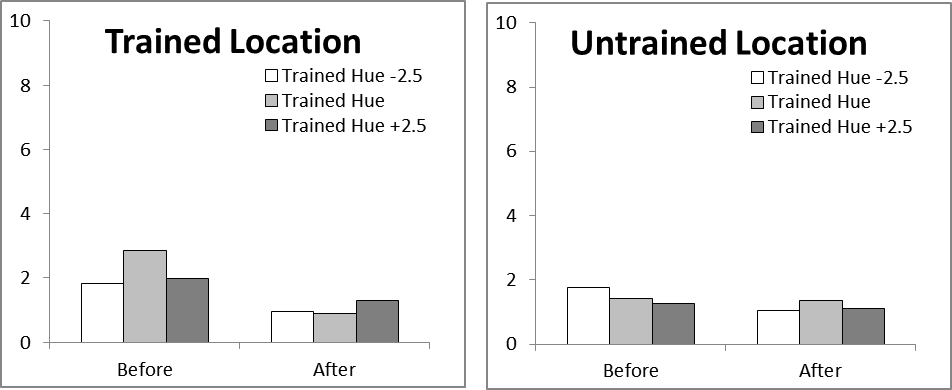


Observer SM – trained location bottom


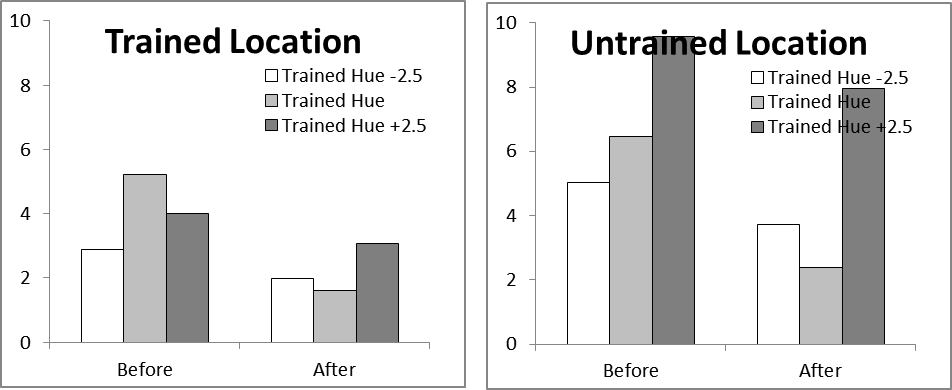


Observer LB – trained location top


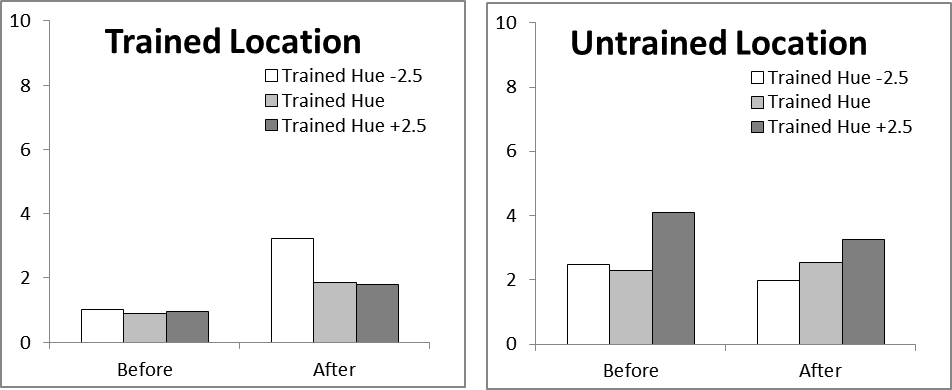


Observer CB – trained location bottom

**
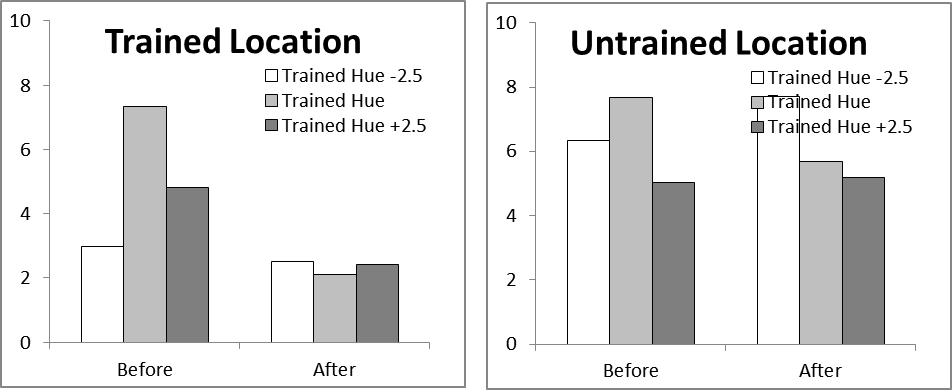
**
